# Neuroendocrine Cells of the Prostate Derive from the Neural Crest*[Fn FN2]

**DOI:** 10.1074/jbc.M116.755082

**Published:** 2016-12-21

**Authors:** Jaroslaw Szczyrba, Anne Niesen, Mathias Wagner, Petra M. Wandernoth, Gerhard Aumüller, Gunther Wennemuth

**Affiliations:** From the ‡ Institute of Anatomy, University Hospital, University Duisburg-Essen, 45147 Essen, Germany,; the § Institute of Pathology, Saarland University Medical School, 66421 Homburg/Saar, Germany, and; the ¶ Department of Anatomy and Cell Biology, Philipps University of Marburg, Robert-Koch-Strasse 8, 35037 Marburg, Germany

**Keywords:** development, neuroendocrinology, neurogenesis, prostate, Wnt pathway, neural crest, neuroendocrine cells, WNT1

## Abstract

The histogenesis of prostatic neuroendocrine cells is controversial: a stem cell hypothesis with a urogenital sinus-derived progeny of all prostatic epithelial cells is opposed by a dual origin hypothesis, favoring the derivation of neuroendocrine cells from the neural crest, with the secretory and basal cells being of urogenital sinus origin. A computer-assisted 3D reconstruction was used to analyze the distribution of chromogranin A immunoreactive cells in serial sections of human fetal prostate specimens (gestation weeks 18 and 25). Immunohistochemical double labeling studies with YFP and serotonin antisera combined with electron microscopy were carried out on double-transgenic Wnt1-Cre/ROSA26-YFP mice showing stable YFP expression in all neural crest-derived cell populations despite loss of Wnt1 expression. 3D reconstruction of the distribution pattern of neuroendocrine cells in the human fetal prostate indicates a migration of paraganglionic cells passing the stroma and reaching the prostate ducts. Double-transgenic mice showed 55% double labeling of periurethral neuroendocrine cells expressing both serotonin and YFP, whereas single serotonin labeling was observed in 36% and exclusive YFP labeling in 9%. The results favor the assumption of a major fraction of neural crest-derived neuroendocrine cells in both the human and murine prostates.

## Introduction

Following secretory and basal cells, neuroendocrine (NE)^2^ cells form the third most common cell type of prostatic epithelium (1–3). NE cells have been regarded as part of the diffuse amine precursor uptake and decarboxylation system (4), elements of which display epithelial, endocrine, as well as neuronal characteristics with nerve-like dendritic processes (5). Two morphological types of prostatic NE cells can be distinguished: the open type, with apical processes reaching to the glandular lumen, and the closed type, with dendrite-like processes between adjacent cells, resting on the basal lamina and without luminal contact (4). The number and distribution of NE cells in the human (3) and rat (6) prostate differs and depends on the location in the gland. In the rat, NE cells are restricted to the periurethral prostatic excretory duct system, whereas in the human gland, both the peripheral glandular system as well as the alveolar glands contain NE cells (1).

As is the case with other cells of the diffuse neuroendocrine system, prostatic NE cells express diverse neurosecretory products such as chromogranin A (CGA) and B (7), serotonin (4), calcitonin family peptides (8) thyroid-stimulating hormone-like peptide (9), bombesin (10), or somatostatin (11). This multitude of secretory products results in a wide diversity of functional subtypes and, thus, in different functions of the prostatic NE cells. Although the physiological functions have not yet been fully understood, it is thought that prostatic NE cells are involved in the regulation, secretion, differentiation, and proliferation of prostatic secretory and basal cells through exocrine, endocrine, paracrine, and autocrine mechanisms. In view of the fact that calcitonin has been found in human seminal fluid, and *in vitro* studies have demonstrated its negative influence on sperm motility, it is feasible that sperm functions may also be the target of NE cells (12, 13). Additionally, these do not express PSA and p63 and are apparently postmitotic and terminally differentiated because of the lack of Ki76 expression (14). Further characteristics of prostatic NE cells are an anti-apoptotic phenotype caused by increased survivin expression (15, 16) and the lack of androgen receptor expression, leading to resistance of NE cell populations in prostatic adenocarcinoma against androgen deprivation therapy and castration (17).

In addition to NE cells in the normal prostate, NE differentiation of prostatic cells into a NE phenotype in prostate cancer is attracting increasing interest as a major factor of diagnostic, prognostic, and therapeutic significance. Although NE cells may be present in carcinoids, small-cell carcinomas are usually entirely or almost entirely composed of tumor cells with NE differentiation (18). NE spots are also encountered in differentiated prostatic adenocarcinoma. NE differentiation is increased in high-grade and high-stage prostatic tumors, and NE tumor cells can promote androgen-independent growth and tumorigenesis (19) as well as invasion and metastasis of prostate cancer cells (20). The origin of NE tumor cells is still not clear, but it is assumed that this cell population shares the same origin with normal prostatic NE cells (21).

The histogenesis of NE cells in the normal prostate has not as yet been fully described, and, up to now, two possibilities of NE cell origin and differentiation have been under discussion. Bonkhoff and Remberger (2) suggested a model describing prostatic stem cells as the clonal origin of NE cells. This assumption is based on the observation that NE cells express basal cell-specific cytokeratins and, thus, could be derived from basal cells in the prostatic epithelium. Furthermore, they postulate that NE cells may also originate from secretory luminal cells because of focal co-expression of PSA and CGA in subsets of NE cells, indicating a derivation from local endodermal cells similar to those of gastrointestinal NE cells (22).

The other possibility discussed would be a neurogenic origin of this cell population (3, 23). We have previously shown the presence of NE cells in prostatic mesenchyme and paraganglia of 10-week-old human embryonic urogenital sinuses, whereas no NE cells were found in the adjacent epithelium. In later development stages, however, NE cells were identified within the epithelial buds, the preliminary stage of glandular structures (23). These findings were interpreted to represent a migration of NE progenitor cells from the neural crest to the developing urogenital sinus, indicating an origin independent of the basal and luminal epithelial cell population. Cassiman *et al.* (24) demonstrated that double-transgenic Wnt1-Cre/ROSA26-YFP mice show stable YFP expression in all neural crest-derived cell populations despite a reduction in Wnt1 expression in neural tube cells during formation of the neural crest. These transgenics are thus suitable for neural crest lineage studies during mouse development. To determine whether prostatic NE cells do, in fact, derive from neural crest cells, we examined Wnt1-Cre/ROSA-YFP mice, which constitutively express YFP in all neural crest-derived cells after Cre-determined excision of a floxed STOP cassette in the ROSA26-YFP locus.

In this paper, we used the knockin mouse model described above to show that prostatic NE cells co-express the murine NE marker serotonin as well as YFP under the control of a WNT1 promoter induced in neural crest-derived cells. Furthermore, 3D reconstruction of serial sections from human fetal prostates labeled with a CGA antibody reveals a pattern of CGA-immunoreactive cells, which suggests the migration of neural crest-derived NE cells in human prostates between the 18th and 25th gestation week (GW). This study confirms migration of neural crest-derived cells to the glandular epithelium of the prostate. Transmission electron microscopy shows that the close contact between murine prostatic NE cells and nerve axons endorses the neurogenic association of prostatic NE cells. With double immunofluorescence, we show that CGA-immunoreactive cells show close proximity to nerve fibers. These findings point to an essential inductive role of neural crest-derived cells during the differentiation of prostatic NE cells.

## Results

### 

#### 

##### Localization of Chromogranin A-positive Cells in the Human Fetal Prostate

**FIGURE 1.**
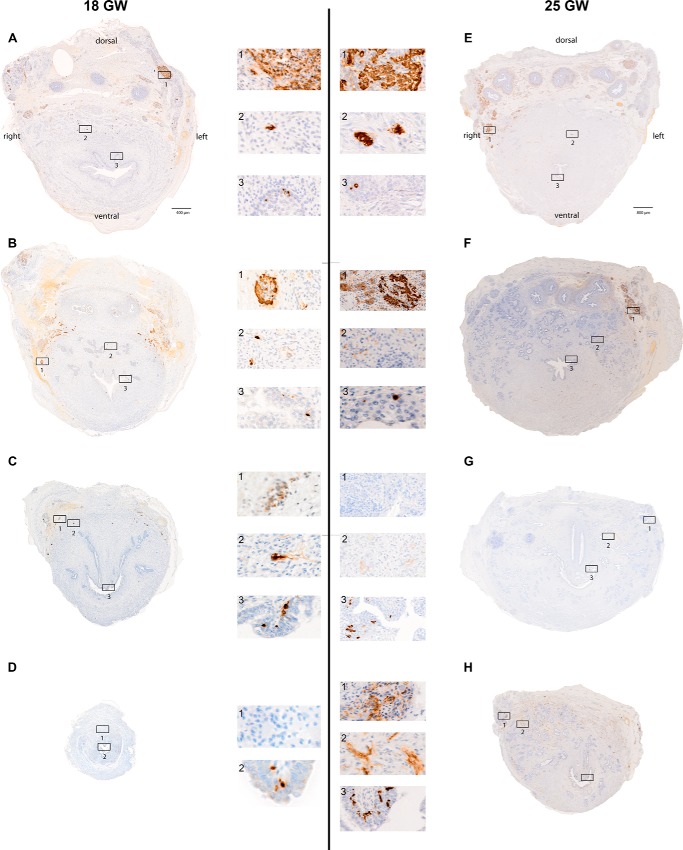
**CGA immunostaining of fetal prostates from gestation weeks 18 and 25.** *A–H*, serial sections of fetal prostates were each divided into quarters from cranial to caudal. Representative stained slices are shown with digitally magnified cutouts (1–3). Prostatic tissue from GW 18 shows CGA-positive cells in the urethral epithelium of all prostate parts (*A3–C3* and *D2*) as well as in prostatic stroma, except in caudal *part D* (*A2–C2*). Additional CGA-positive cells are situated in distinct paraganglionic structures of *parts A–C* (*A1–C1*). In GW 25, positive signals are present in the urethral epithelium (*A3–D3*) and paraganglia (*A1*, *B1*, and *D1*), comparable with GW 18. In contrast to GW 18, there are only few CGA-positive cells in stromal tissue, situated in the cranial part of the prostate (*E2*), but more axon-like structures are detectable (*G2* and *H2*).

We investigated the localization of NE cells in serial slices of human fetal prostates from GW 18 (800 slices) and 25 (1200 slices) using an anti-CGA antibody as an immunohistochemical marker and diaminobenzidine (DAB) as chromogen. CGA is a generally accepted NE cell marker that is expressed in NE cells during fetal development of several human tissues, *e.g.* pancreas or lung (25, 26). The CGA-positive cells in fetal prostates are located in the epithelial compartment with proximity to nerve fibers, as typically described for prostatic NE cells. Therefore, we assume that most of the CGA-immunoreactive cells are NE cells. We found that most of the openings of the prostate gland ducts are located in the caudal part of the urethra. Therefore, serial slices were divided into four craniocaudally oriented horizontal portions (I–IV) to describe their distribution, and representative reaction results from the center of each part are shown (Fig. 1). CGA-positive cells could be identified in all parts of both prostate specimens following the longitudinal axis of the organ. This cell population is present in extraprostatic paraganglia in the form of distinct cell foci (Fig. 1, *A1–H1*) as well as in urethral and prostatic epithelium (Fig. 1, *A3–H3*). Additionally, CGA-positive cells were found in prostatic stroma of cranial prostate parts of the specimen of GW 18 (Fig. 1, *A2–H2*). In the specimen of GW 25, an increasing number of axonal structures in the prostate can be observed (Fig. 1, *F2* and *H2*), whereas there is a decrease in the number of both stromal and paraganglionic CGA-positive cells. The increasing number of CGA-positive cells in the epithelium correlates with the progressive development of glandular structures during prostate development (Fig. 1, *F–H*).

##### Changes in NE Cell Distribution in the Fetal Prostate during Embryogenesis from Gestation Week 18 to 25

**FIGURE 2.**
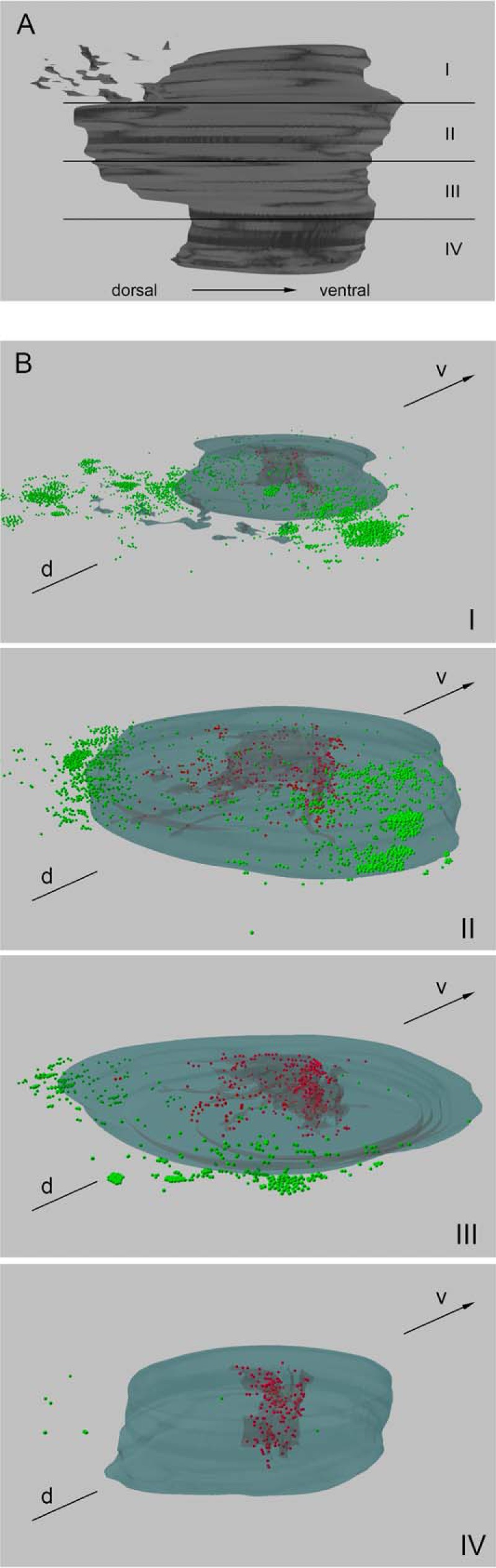
**3D reconstruction of serial slices and distribution of CGA-positive cells in fetal prostate from GW 18.** *A*, 3D model of a fetal prostate from GW 18 containing 73 slices, viewed from the right. *I–IV* indicate the subdivisions of the prostate model displayed in the lower pictures. *B*, the majority of NE cells in cranial *parts I* and *II* are situated outside of the prostate in extraprostatic paraganglia (*green*). In the prostate, *part I* shows more NE cells in the stroma (*green*) than in urethral and glandular epithelium (*red*) compared with *part II*, which are arranged in diagonal pathways. The number of NE cells outside of the prostate continuously decreases in the caudal direction, conversely to epithelial NE cells (*III* and *IV*). *d*, dorsal; *v*, ventral.

**FIGURE 3.**
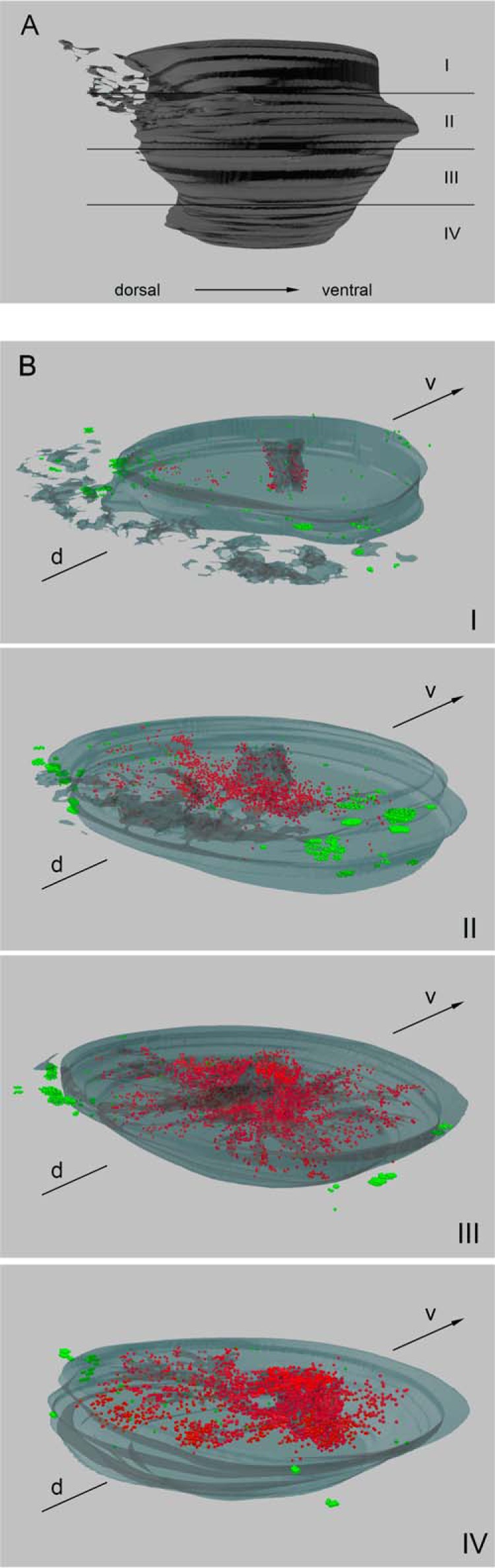
**3D reconstruction of serial slices and distribution of CGA-positive cells in fetal prostate from GW 25.** *A*, 3D model of a fetal prostate from GW 25 containing 108 slices, viewed from the right. *2–5* indicate the subdivision of the prostate model displayed in the *bottom pictures. B*, the number of CGA-positive cells in extraprostatic paraganglia and prostatic stroma (*green*) decreases in comparison with GW 18. *d*, dorsal; *v*, ventral.

To assess the changes in the distribution of NE cells during prostate development and, thus, to find indications for potential NE cell migration, we reconstructed a 3D model from the stained serial slices described above. Briefly, each tenth slice was scanned as a whole, and the pictures obtained were formatted and inserted into BioVis 3D software. After manual highlighting of structures and CGA-positive cells, the models created were again divided into four transparent parts (Figs. 2*A* and 3*A*) and rotated.

The prostate model from GW 18 shows an unequal distribution pattern of epithelial and stromal/extraprostatic NE cells (Fig. 2*B*). In the top cranial part, the majority of CGA-positive cells are present in paraganglia and prostatic stroma (Fig. 2*B*, *green dots*), which are arranged diagonally to the dorsoventral axis toward the distal ends of the U-shaped urethra (Fig. 2*B*, *I*, and supplemental Movie 1). In the more caudal parts the number of stromal and extraprostatic NE cells decreases. The second part shows a decline of this NE cell population down to 50% (Fig. 2*B*, *II*, and supplemental Movie 2), in the third part there is a reduction of 91% (Fig. 2*B*, *III*, and supplemental Movie 3), and in the most caudal part there are hardly any stromal or extraprostatic NE cells to be found (Fig. 2*B*, *IV*, and supplemental Movie 4). Only a few NE cells are detectable in prostatic and urethral epithelium, where *red dots* (Fig. 2*B*) represent NE cells in the epithelium of glandular structures and the urethra. Because of the fact that there are not many glandular structures at this stage of development, epithelial CGA-positive cells are mostly situated in the urethral epithelium.

In contrast, GW 25 presents a different pattern of NE cells. Especially in the upper half of the prostate, a decline of extraprostatic as well as stromal CGA-positive cells is observed (Fig. 3*B*, *I* and *II*, and supplemental Movies 5 and 6) compared with epithelial NE cells. The majority of extraprostatic NE cells are situated in the second upper part of the prostate (Fig. 3*B*, *II*), containing three to four times more NE cells than the adjacent parts (Fig. 3*B*, *I* and *III*) but showing only few paraganglionic structures with CGA staining. The distribution pattern is comparable with GW 18 with diagonal positions. In contrast, the prostatic and urethral epithelia show an increase in NE cell number in the three lower parts. The majority of CGA-positive cells are arranged in the distal ends of the urethral epithelium and in prostatic glands (Fig. 3*B*, *III* and *IV*, and supplemental Movies 7 and 8).

##### Accumulation of NE Cells in the Caudal Prostate from Gestation Week 18 to 25

**FIGURE 4.**
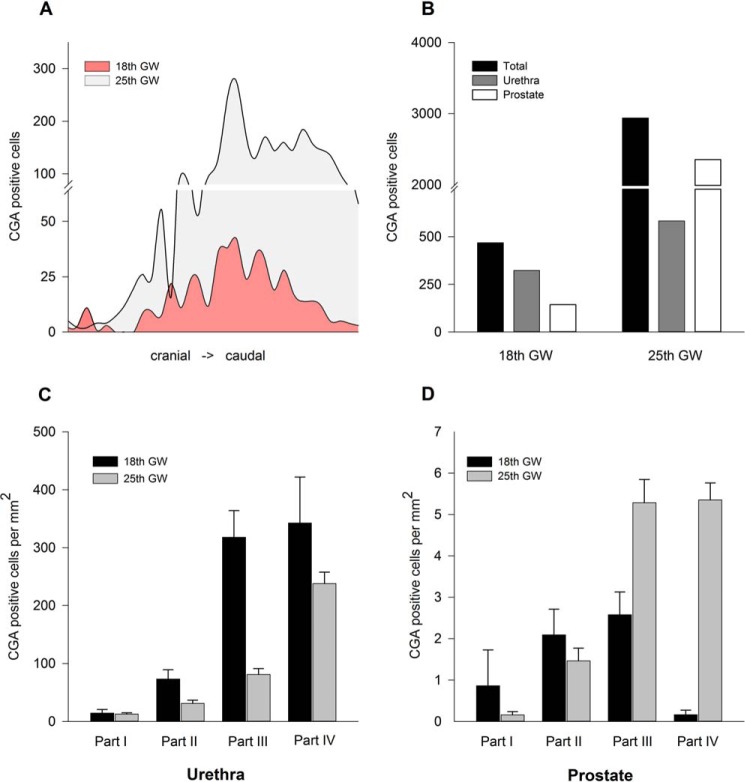
**Distribution and density of NE cells in fetal prostates from GW 18 and 25.** *A*, the distribution of the total NE cell population in GW 18 and 25 shows a similar pattern, in which the central prostatic part contains the majority of CGA-positive cells, with 274 CGA-positive cells per slice in GW 25 and 42 CGA-positive cells in GW 18. *B*, the total number of NE cells in GW 25 is considerably higher than in GW 18. It rises in total from 470 to 2940 cells, counted in the corresponding 32 slices. The number of NE cells in prostatic tissue is 16 times higher in the later development stage but almost doubled in the urethral epithelium. *C*, in both developmental stages, the density of CGA-positive cells in the urethra increases in the caudal direction. In GW 25, the cell density significantly decreases in *part I* from 73.2 to 31 cells/mm^2^ (*p* = 0.0129) and in *part II* from 318 to 81 cells/mm^2^ (*p* < 0.001) compared with GW 18. *D*, in contrast to the urethra, the density of NE cells in the caudal prostate in GW 25 amounts to 5.3 and 5.4, respectively, and is significantly higher (*p* < 0.001) compared with GW 18, whereas no differences in NE cell density are detectable in cranial *parts I* and *II*. In *C* and *D*, mean values ± S.E. from eight slices are shown.

As the 3D reconstruction of the fetal prostates revealed developmental changes in NE cell distribution, our goal was to quantify NE cell density in both prostates. For this purpose, eight slices from each of the four parts of the respective prostate were chosen at determined intervals, and the total number of NE cells in the 32 slices was determined. Fig. 4*A* shows the number of NE cells in each slice in a cranial-to-caudal course. Both prostates show a similar distribution of NE cells in the prostate, with a maximum of 274 CGA-positive cells per slice in GW 25 and 42 CGA-positive cells in GW 18 in the central part. The total number of NE cells in the 32 slices increases from GW 18 to GW 25 from 467 to 2936 cells (Fig. 4*B*). After discrimination of NE cell localization between urethral epithelium and prostatic tissue, further distributional differences appear. The majority of NE cells in GW 18 are localized in the urethral epithelium and are nearly doubled from 323 to 582 in GW 25, whereas the number of prostatic NE cells is 16 times higher (2354 compared with 144), which represents 80% of NE cells identified in the later stage of development.

Fig. 4, *C* and *D*, shows the relative NE cell density after an additional discrimination of urethral epithelium and prostatic buds along a cranial-caudal axis in relation to the respective area. In urethral epithelium, NE cell density remains low in cranial part I, with 14.4 ± 6.4 and 12.7 ± 2.2 cells/mm^2^ during development from GW 18 to GW 25 but significantly decreases from 73.2 ± 15.9 to 31 ± 5.7 cells/mm^2^ (*p* = 0.0129) in part II and from 318 ± 45.9 to 81 ± 10.1 cells/mm^2^ (*p* < 0.001) in caudal part III. Part IV shows the highest cell density in both developmental stages, with 342.5 ± 79.5 and 237.9 ± 19.9 cells/mm^2^ (Fig. 4*C*). In contrast, NE cell density in prostatic bud tissue does not show any significant changes in cranial parts I and II but does increase in caudal part III from 2.6 ± 0.6 to 5.3 ± 0.6 cells/mm^2^ (*p* < 0.001) and displays the most considerable difference in part IV, with a rise from 0.2 ± 0.1 to 5.4 ± 0.4 cells/mm^2^. Statistical analysis reveals a shift of NE cell density from urethral to prostatic epithelium in more caudal parts during prostatic development between GW 18 and GW 25.

##### A Subset of Prostatic NE Cells in Wnt-1/YFP Knockin Mice Co-expresses Serotonin and YFP

**FIGURE 5.**
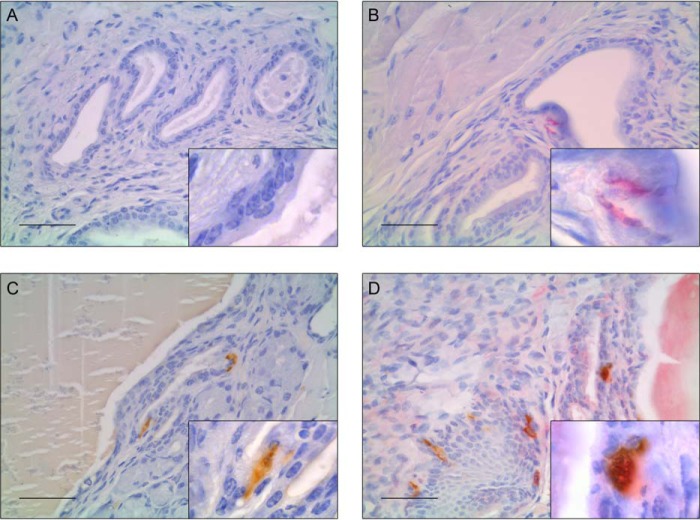
**Immunohistochemical double staining of serotonin and YFP in Wnt1-Cre/ROSA26-YFP mice.** Urogenital tracts from Wnt1-YFP double knockin mice containing urethra and prostate lobes were embedded, and the respective slices were incubated with primary antibodies against serotonin, YFP, or both. The color reaction was performed using DAB (*brown*) for serotonin and Vector® Red for YFP (*magenta*) as chromogens. *B* and *C*, selective staining for either serotonin or YFP shows the respective expression in epithelial cells of prostatic glandular ducts. *D*, double staining of the corresponding tissue reveals cells showing co-expression of serotonin and YFP. Negative controls without primary antibody did not exhibit any signal (*A*). *Scale bars* = 50 μm.

To determine whether prostatic NE cells derive from neural crest cells, we examined Wnt1-Cre/ROSA-YFP mice, which constitutively express YFP in all neural crest-derived cells after Cre-determined excision of a floxed STOP cassette in the ROSA26-YFP locus (24). With this approach, both serotonin and GFP antibodies were used for immunohistochemical double labeling of the corresponding urogenital tract, in particular of the zones containing the apertures of the prostatic gland ducts. Single labeling for serotonin in prostatic and urethral tissue showed the usual NE cell distribution, with the majority of cells situated in the periurethral epithelium of prostatic glandular duct structures (Fig. 5*C*). YFP expression was detected in identical areas (Fig. 5*B*). Additionally, nerve axons adjacent to the prostatic alveoli distant from the outside of the urethra showed strong YFP expression. Double labeling for serotonin and YFP revealed cells expressing both serotonin and YFP (55% of immunoreactive cells) as well as cells with only serotonin (36%) or YFP (9%) signals, respectively, indicating that approximately a little more than half of the prostatic NE cells displayed the neural crest marker (Fig. 5*D*). This is a crucial finding that needs detailed discussion.

##### CGA-immunoreactive cells Show Close Proximity to Nerve Fibers in Mouse and Human

**FIGURE 6.**
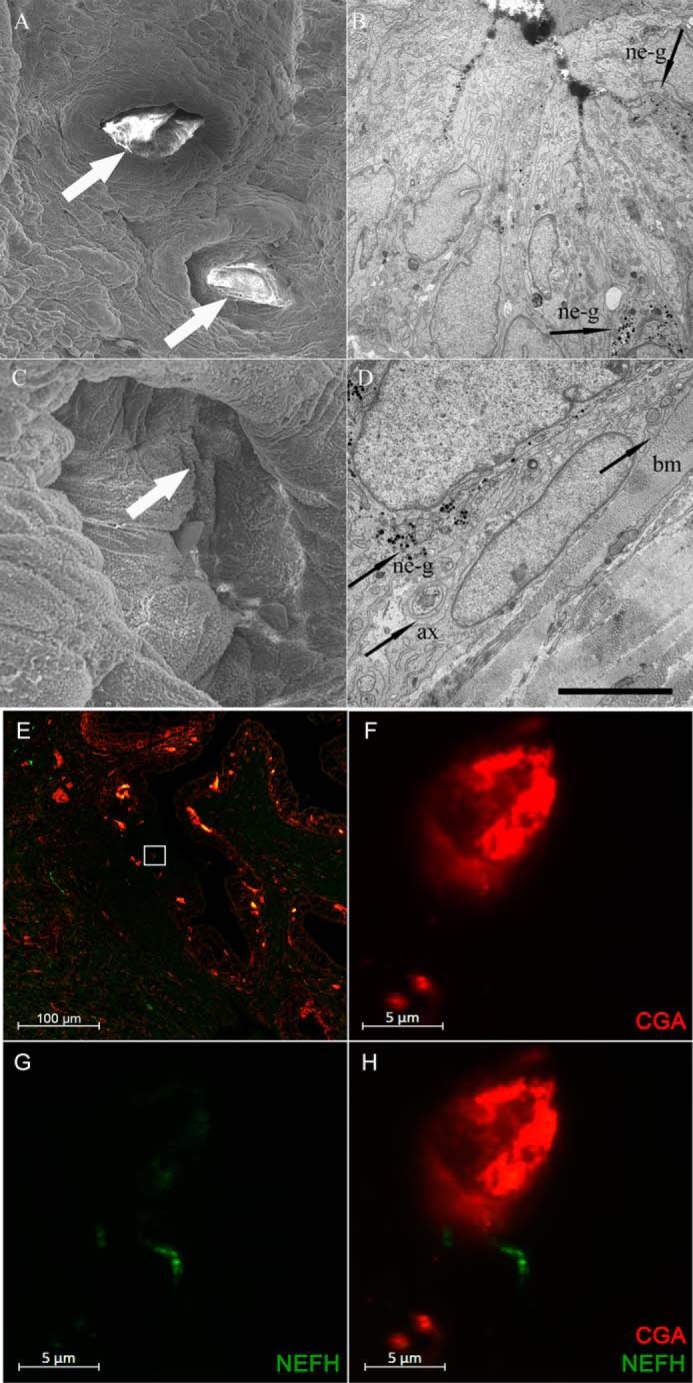
**Electron microscopy of murine prostatic NE cells and immunofluorescence double staining of CGA and neurofilament heavy chain (NEFH) in human fetal prostate from GW 25.** *A–D*, scanning (*A* and *C*) and transmission (*B* and *D*) electron micrographs of murine urethral surface epithelium and openings of ventral prostate gland ducts. *A*, survey scanning electron micrograph of urethral mucosa and openings of gland ducts (*arrows*) containing clotted secretion. *Scale bar* = 50 μm. *B*, NE cells of the closed (*bottom margin*, *right*, *arrow*) and open (*right margin*, *top right*, *arrow*) type containing typical granules (*ne-g*) in a terminal prostatic duct. *Scale bar* = 10 μm. *C*, opening of a secretory duct (*arrow*). In this region, NE cells of the open type are regularly present. *Scale bar* = 10 μm. *D*, basal portion of a ductal NE cell resting on the basal lamina of the basal membrane (*bm*). Note the close relationship of the granule-containing (*ne-g*) portion of the cell and a nerve axon. *Scale bar* = 5 μm. *E–H*, double-immunostaining of CGA (*red*) and NEFH (*green*) in human fetal prostate from GW 25. *E*, overview of double-stained fetal prostatic tissue. Most CGA signals are situated in the glandular epithelium. The *white rectangle* marks a magnified cell from *F–H. F*, the depicted CGA-positive NE cell is situated in the glandular epithelium and shows a close proximity to a nerve fiber (*G* and *H*).

Scanning electron microscopy of the urethral openings of the ventral prostate excretory ducts showed homogeneous epithelial surfaces and an absence of the microvilli-studded cell apices of NE cells, confirming the extremely low frequency of open-type NE cells in this area (Fig. 6, *A* and *C*). Transmission electron microscopy revealed a clear-cut prevalence of NE cells containing typical serotonin granules located basally in the epithelium and in the close vicinity of stromal nerve axons (Fig. 6, *B* and *D*). Small granule cells and direct nerve contacts, however, were extremely rare, reflecting a similar situation as found in the rat. Double immunolabeling of human fetal prostate from GW 25 shows that nerve fibers are closely located near CGA-immunoreactive cells (Fig. 6, *F–H*) and implies that innervation of NE cells starts at an early stage of maturation.

## Discussion

Using 3D reconstructions of two developmental stages of human prostate specimens, from GW 18 and 25, respectively, we showed characteristic distribution patterns of CGA-labeled NE cells, confirming earlier semiquantitative studies (3, 23). The 18-week specimen contained labeled cells in the (peri)prostatic paraganglia and cranial prostatic stroma and in epithelial buds of prostatic anlagen, originating from the urethral epithelium. There was an obliquely oriented gradient in NE cell density, with the highest cell count in the cranial stroma (along with the paraganglia) and only few cells in prostate gland anlagen, but a higher density in the urethral epithelium. In the specimen taken from GW 25, the distribution pattern had shifted into the direction of the prostatic gland buds, with only few stromal NE cells left. The change of distribution on NE cells between cranial and caudal parts of the urethra might affect the presence of NE cells in transitional, proximal, or central zones of the human prostate in adults. Regarding this question, we cannot make any conclusions because in our study we used tissue of GW18 or 25 only, where the adult zonal differentiation is not yet recognizable. In view of the close vicinity of immunoreactive axons and cells, it seems likely that nerve axons, sprouting from the paraganglia, pierce the prostatic stroma and serve as tracks for the migrating NE cells.

A comparison of both developmental stages shows that the overall number of prostatic NE cells increased by approximately a factor 6, from 467 up to 2936. No CGA-labeled cells whatsoever were found either in the stroma or in the epithelium of the seminal vesicles and ejaculatory ducts. These figures indicate a migration process of NE cells from the neural crest-derived (peri)prostatic paraganglia via prostatic stroma into prostatic gland buds and the urethral epithelium. Although ejaculatory ducts and seminal vesicles are derived from the mesodermal wolffian duct, the prostate gland buds are of endodermal origin, originating in the urogenital sinus. Hence, the different origin of both structures provides a signal for the migrating NE cells to avoid the wolffian duct-derived structures and to attach to the urogenital sinus-derived elements. The significant increase in NE cells during migration, and particularly in the prostatic epithelium, presumably indicates a differentiation process associated with migration. Additionally, recruitment of stem cells by priming of prostatic cells through cell-to-cell interaction of neuroendocrine and urogenital sinus-derived cells is conceivable.

To determine the significance of neural crest-derived cells in the histogenesis of NE cells, we chose the double knockin model of Cassiman *et al.* (24). Prior to commencement, the suitability of the mouse urogenital tract as a model for human prostatic NE cells had to be verified. As had been shown previously for the rat urogenital tract (6), the distribution pattern of murine prostatic NE cells differs from the situation in humans in that, in the former, the NE cells are generally absent from the prostatic glandular epithelium proper and restricted to the terminal gland ducts of the ventral (and less of the lateral and dorsal) prostate and the urethral opening of the terminal excretory duct of the seminal vesicle (even though the definite topographical situation is controversial (27)).

In the human prostate, NE cells are present both in periurethral excretory ducts (at higher density) as well as scattered in peripheral acini. They are absent from the anterior prostate, ejaculatory ducts, and seminal vesicles (28). As has been shown by immunohistochemistry and scanning and electron microscopy, rat and mouse prostatic NE cells show identical characteristics, the most common type being the serotonin-containing closed type, situated in the basal epithelium of prostatic excretory ducts, often in the vicinity of stromal nerve axons. We show that, in humans, the innervation of such NE cells starts before GW 25. The use of the knockin mouse model of Cassiman *et al.* (24) to study the origin of prostatic NE cells involved the observation of a specific labeling pattern, which requires explanation. Approximately 55% of the murine prostatic NE cells were double-labeled for serotonin and the YFP-labeled neural crest marker. This clearly indicates that these cells originated from the neural crest during embryonic development. In 9% of the NE cells, however, YFP labeling was exclusively observed, whereas in 36%, only serotonin immunoreactivity was detected, and no YFP labeling was observed.

A possible explanation for the lack of serotonin expression in 9% of YFP-positive cells is the diversity of neurosecretory products expressed by NE cells, whose implication is still discussed and may result in subpopulations of NE cells with varying combinations of secretion products (4, 7–11) or in different functional states of single NE cells. In total, 64% of the YFP-labeled prostatic NE cells clearly indicate being derived from the neural crest. There are two possible reasons why this percentage is not higher. Most likely, it is the recombination efficiency in Wnt1 expression in neural crest-derived cells, which is never 100%, and also, it depends on the strength of reporter expression and the method of visualization (in this case, the use of an anti-GFP antibody). More recently developed fluorescent markers, such as R26-td Tomato, as used in Ref. 29 in their study on the embryonic origin of thyroid C cells in mice and humans, have a higher labeling efficiency. A less likely possibility would be that the Rosa locus, even though being ubiquitously expressed before birth, may not be well detected in some adult NE cells when using the YFP visualization system. Therefore, the 36% of YFP-negative cells could lack the signal for different reasons; for instance, different functional or maturation stages, as is true for neural crest-derived cutaneous melanoblasts and Merkel cells (30). Also, it is conceivable that they represent different cell types, such as the recently described urethral sensory brush cells (31), the origin of which is controversial (32).

Cell migration is common to neural crest-derived precursor cells, the best example being cutaneous melanoblasts. In a comparison of prostatic NE cells with cutaneous melanoblasts (33–41), the more likely explanation for the different forms observed would seem to be the developmental change in gene expression. During colonization of the epidermis by melanoblasts, substantial proliferation occurs, generating a plethora of cells from a few founders and controlled by intrinsic and extrinsic factors (33–41). They are required for tissue-specific stem cells to arise from lineage-specified precursors, including the regulation of transcription factors important in defining the specific lineage (37). The melanoblast system permits the study of *in vitro* interaction of neural crest-derived cells with ectodermal keratinocytes, thereby enabling identification of the factors responsible for the migration route (33), proliferation, and differentiation intensities of the precursor cells. Among these factors, not only have specific molecules been identified, guiding the migration route, but also factors such as stem cell factor, BMP4, (38) have been identified along with cell-cell-interactions, regulating gene expression during lineage-specific differentiation of cutaneous melanocytes (33–35). Similar mechanisms could also be active during the colonization of prostatic epithelium by neural crest-derived precursors of NE cells, showing different patterns of gene expression. Earlier we stated (6) that the invasion of prostatic epithelium by NE cells had not been directly observed, which leaves two possibilities open: either neural crest-derived NE cells are only required to trigger neuroendocrine development in individual prostatic stem cells, or precursor cells lacking CGA or serotonin immunoreactivity enter the prostatic epithelium, where they differentiate into mature serotonin-containing prostatic NE cells. Prostatic NE cells showing both serotonin and YFP immunoreactivities along with smaller fractions featuring either single serotonin or single YFP immunoreactivity point to the latter possibility.

## Experimental Procedures

### 

#### 

##### Human Prostate Specimens

Human prostates taken from routine post mortems of legal abortion material from gestation weeks 18 and 25 were provided by the Department of General and Special Pathology, Saarland University, Homburg/Saar. This study was approved by the local ethical review board (no. 145/11, Saarland Ethical Commission). Specimens had been fixed with Bouin's fluid and paraffin wax-embedded.

##### Animals and Tissue Preparation

Wnt1-CRE mice expressing CRE recombinase enzyme under the control of a Wnt1 promotor/enhancer construct and ROSA26/YFP reporter mice carrying a YFP sequence preceded by a floxed STOP cassette in the ROSA26 locus were kindly donated by Dr. Christo Goridis (Département de Biologie, École Normale Supérieure, Paris, France) and were crossed to generate heterozygous Wnt1-CRE/YFP mice. Polymerase chain reaction was used to determine CRE and YFP expression in double-transgenic Wnt1-CRE/YFP mice. All male transgenic animals (aged 14–16 weeks) were perfusion-fixed with 4% paraformaldehyde in PBS buffer through flushing of the vascular system via the left ventricle after severing of the right atrium of the heart. Following perfusion, the urogenital tract containing the bladder, urethra, and the prostatic lobes as well as the adrenal glands were excised and further fixed overnight in PBS-buffered 4% paraformaldehyde solution followed by a washing step in 70% ethanol for 24 h.

##### Immunohistochemistry

Paraffin-embedded tissue blocks were cut at a thickness of 7 μm to obtain serial sections in coronal orientation of the prostate. Slices were mounted on glass slides, deparaffinized with xylene, and rehydrated in a descending alcohol series (100%, 90%, 80%, and 70%).

For indirect immunoperoxidase reactions, slices were washed three times for 5 min in PBS and once for 15 min in PBST (5% Tween 20 in PBS) and blocked for 30 min at room temperature with 5% BSA in PBST. Slices were incubated overnight at 4 °C with rabbit anti-CGA (ab15160, Abcam, Cambridge, UK) and diluted 1:400 in PBS with 5% BSA. Murine urogenital tract slices were equally incubated with goat anti-serotonin antibody (ab66047, Abcam) diluted 1:200. After three washing steps of 15 min in PBST and an additional blocking step, the slices were incubated for 1 h at room temperature with the corresponding secondary biotinylated rabbit anti-goat IgG or goat anti-rabbit IgG (BA-5000/BA-1000, Vector Laboratories, Burlingame, CA) diluted 1:200/1:400 in PBS/5% BSA, respectively. Finally, the slices were washed three times for 15 min with PBST and 5 min with PBS.

Signal enhancement was achieved by using the Vectastain® kit (Linaris, Wertheim-Bettingen, Germany) according to the protocol of the manufacturer. Immunoreaction was visualized using diaminobenzidine (DAB) (Sigma) as a chromogen. As a control, incubation without the primary antibody or with a nonspecific serum was also performed. Nuclear staining was carried out by treating the slides for 1 min with hemalum (Roth, Karlsruhe, Germany), followed by 5-min incubation in tap water to induce the color reaction. Eventually, the stained slices were dehydrated and mounted with DEPEX (Serva, Heidelberg, Germany). A light microscope (Axiophot, Zeiss, Jena, Germany) was used for evaluation.

For immunoreactions using alkaline phosphatase-linked labeling, several changes in the protocol had to be observed. The first blocking step was carried out for 45 min at 37 °C with glucose oxidase (Sigma) in PBS-glucose buffer (10 mm glucose, 1 mm NaN_3_, and 0.4 units/ml glucose oxidase) to disable endogenous peroxidase activity. The antibodies used were chicken anti-GFP (Aves Labs, Tigard, OR) diluted 1:1200 in PBS with 5% BSA and avidin (1:300, Merck) and goat anti-chicken IgY (Aves Labs) diluted 1:200 in PBS with 5% BSA and biotin (1:50, Sigma). As an anti-GFP antibody provides considerably more intensive signals than an anti-YFP antibody, this was used for histochemical localization of YFP in combination with the Vectastain ABC-AP kit (Vector Laboratories, Burlingame, CA).

##### Double Immunofluorescence

After rehydration, antigens were unmasked twice with citrate buffer, and slices were washed with PBS/0.025% Triton X-100 for 10 min and blocked for 30 min at room temperature with PBS/5% donkey serum. Subsequently slices were incubated overnight at 4 °C with chicken anti-NEFH (ab4680, Abcam) diluted 1:5 in PBS with 2% donkey serum. After two rinses in PBS, slices were incubated for 1 h at room temperature with donkey anti-chicken Alexa 488 antibody (703-545-155, Dianova, Hamburg, Germany) diluted 1:100 in PBS. After two additional washing steps with PBS, slices were blocked for 30 min at room temperature with 5% goat serum and incubated overnight at 4 °C with rabbit anti-CGA (ab15160, Abcam) diluted 1:100 in PBS/2% goat serum. After two rinses in PBS, slices were incubated for 1 h at room temperature with goat anti-rabbit Alexa 568 antibody (A-11036, Thermo Fisher Scientific, Schwerte, Germany) diluted 1:200 in PBS, washed twice in PBS, and mounted with Fluoromount (Sigma-Aldrich, Munich, Germany). For signal detection, a Zeiss ELYRA PS.1 structured illumination microscope system was used.

##### Electron Microscopy

Following vascular perfusion fixation of the adult rats and mice as described, the male accessory sex organs were isolated, and the prostatic urethra (of one rat) was cut transversely into five portions (1, below the bladder; 5, retropubic portion), each about 2 mm thick. Prostate lobes, seminal vesicles, and coagulating glands taken from other specimens were cut into small pieces, and all samples were post-fixed in 1% osmium solution in sodium cacodylate buffer (0.1 m, pH 7.5) for 1 h. Following careful washing in the same buffer, dehydration was performed in a graded series of ice-cold ethanols and propylene oxide, and penetration was performed with a propylene oxide-epon mixture for 24 h. Specimens were then transferred into a fresh epon mixture and polymerized for 24 h at 56 °C. The blocks were trimmed as usual, and semithin sections were cut on an ultramicrotome and stained with toluidine-fuchsine solution. Appropriate areas were selected, trimmed, and cut with a diamond knife. Silver-to-gray ultrathin sections were treated with lead citrate and uranyl acetate solutions and examined under a Zeiss EM-10C electron microscope.

For scanning electron microscopy, murine urethrae from adult male NMRI mice were taken from perfusion-fixed animals (for details of the methodology, see Aumüller *et al.* (6)) after dissecting the accessory sex glands by removing the pubic bones and cutting the urethra directly underneath the bladder orifice and above the urethral bulb. The urethral lumen was opened through a medial incision, and the remaining seminal clotted material was flushed away using a syringe jet wash with PBS. Specimens were dehydrated, critical point-dried, gold-coated according to standard procedures, and examined in a scanning electron microscope.

##### 3D Reconstruction

Each tenth CGA-labeled slice from the human prostate serial sections was whole-scanned using an Olympus BX51 microscope system. After file formatting, the pictures obtained were inserted in BioVis3D reconstruction software. All dominant glandular structures, the urethral lumen, as well as positively stained cells were marked manually. Visualized cells situated in the epithelium of the urethra or in the prostatic ducts were marked in red and cells in stromal or extraprostatic tissue in green. The reconstructed 3D model of human fetal prostates contained 73 single pictures for gestation week 18 and 108 single pictures for gestation week 25.

##### Statistical Analysis

For statistical analysis of NE cell distribution in the human fetal prostates, both serial sections were split into four parts (cranial, medium cranial, medium caudal, and caudal), each containing eight slices at distinct gaps. The number of CGA-positive cells for each slice and the resulting mean for each part were determined. To estimate the relative NE cell distribution and the NE cell density in the urethra and prostatic tissue, the area of urethral epithelium and extraurethral tissue of all analyzed slices was defined by measuring the number of pixels in the corresponding areas. In the case of normal distribution, Student's *t* test was performed to determine statistical significance, with *p* < 0.05. Apart from that, Mann-Whitney rank-sum test was carried out.

##### Software

Documentation and analysis of agarose gels were performed with Bio-Rad Image Lab. Serial sections from human prostates were scanned using dotSlide 2992 software (Olympus, Mt. Waverley, VIC, Australia). Adobe Photoshop CS6 (San Jose, CA) was used for size and orientation formatting of the obtained pictures. 3D reconstruction was carried out with BioVis3D reconstruction software (BioVis3D, Montevideo, Uruguay). Statistical analyses were performed with SigmaPlot 13.5 (Systat, San Jose CA).

## Author Contributions

J. S., G. W., G. A., M. W., P. W., and A. N. performed the experiments. J. S., G. W., and G. A. wrote the manuscript.

## Supplementary Material

Supplemental Data

## Footnotes

2 The abbreviations used are:

NE: neuroendocrine
CGA: chromogranin A
DAB: diaminobenzidine
GW: gestation week
NEFH: neurofilament heavy chain.
