# Supplementary material for: Neuroendocrine Cells of the Prostate Derive from the Neural Crest
Source: J Biol Chem. 2016 Dec 21;292(5):2021–31. doi: 10.1074/jbc.M116.755082 (PMC5290971; doi:10.1074/jbc.M116.755082)
Supplement: Supplemental Data [file supp_292_5_2021__index.html]

Neuroendocrine cells of the prostate derive from the neural crest — Neuroendocrine Cells of the Prostate Derive from the Neural Crest — Derivation of Neuroendocrine Cells in the Prostate — Supplemental Data 

# Neuroendocrine Cells of the Prostate Derive from the Neural Crest

## Supplemental Data

- Supplemental Movie 1 (.mov, 26.2 MB) - distribution of CGA-positive cells in the top cranial part of fetal prostate from GW 18
- Supplemental Movie 2 (.mov, 19.6 MB) - distribution of CGA-positive cells in the second upper part of fetal prostate from GW 18
- Supplemental Movie 3 (.mov, 24.8 MB) - distribution of CGA-positive cells in the third part of fetal prostate from GW 18
- Supplemental Movie 4 (.mov, 9.7 MB) - distribution of CGA-positive cells in the lowermost part of fetal prostate from GW 18
- Supplemental Movie 5 (.mov, 23.5 MB) - distribution of CGA-positive cells in the top cranial part of fetal prostate from GW 25
- Supplemental Movie 6 (.mov, 30.7 MB) - distribution of CGA-positive cells in the second upper part of fetal prostate from GW 25
- Supplemental Movie 7 (.mov, 32.0 MB) - distribution of CGA-positive cells in the third part of fetal prostate from GW 25
- Supplemental Movie 8 (.mov, 24.6 MB) - distribution of CGA-positive cells in the lowermost part of fetal prostate from GW 25
